# Protein Kinase D1 regulates focal adhesion dynamics and cell adhesion through Phosphatidylinositol-4-phosphate 5-kinase type-l γ

**DOI:** 10.1038/srep35963

**Published:** 2016-10-24

**Authors:** Nisha Durand, Ligia I. Bastea, Jason Long, Heike Döppler, Kun Ling, Peter Storz

**Affiliations:** 1 Department of Cancer Biology, Mayo Clinic, Jacksonville, Florida 32224, USA; 2 Department of Biochemistry and Molecular Biology, Mayo Clinic, Rochester, Minnesota 55905, USA

## Abstract

Focal adhesions (FAs) are highly dynamic structures that are assembled and disassembled on a continuous basis. The balance between the two processes mediates various aspects of cell behavior, ranging from cell adhesion and spreading to directed cell migration. The turnover of FAs is regulated at multiple levels and involves a variety of signaling molecules and adaptor proteins. In the present study, we show that in response to integrin engagement, a subcellular pool of Protein Kinase D1 (PKD1) localizes to the FAs. PKD1 affects FAs by decreasing turnover and promoting maturation, resulting in enhanced cell adhesion. The effects of PKD1 are mediated through direct phosphorylation of FA-localized phosphatidylinositol-4-phosphate 5-kinase type-l γ (PIP5Klγ) at serine residue 448. This phosphorylation occurs in response to Fibronectin-RhoA signaling and leads to a decrease in PIP5Klγs’ lipid kinase activity and binding affinity for Talin. Our data reveal a novel function for PKD1 as a regulator of FA dynamics and by identifying PIP5Klγ as a novel PKD1 substrate provide mechanistic insight into this process.

Focal adhesions (FAs) are specialized attachment sites that connect cells to the extracellular matrix (ECM)^1^, transmit mechanical force and relay regulatory signals across the cell-ECM interface^2^,^3^. FAs are dynamic structures that can influence multiple aspects of cell behavior including adhesion, motility and proliferation^4^. To effectively elicit their biological functions, FAs need to be assembled and disassembled continually. For example, a crucial phase of the motile cycle involves the assembly of new FAs at the leading edge and disassembly of FAs towards the rear of the cell which facilitates translocation of the cell body^5^. The turnover of FAs (assembly and disassembly) is a highly coordinated process that is regulated by a complex network comprising of Rho GTPases, adaptor proteins, kinases and phosphatases^6^,^7^,^8^,^9^.

Initial FA formation is mediated by integrins; heterodimeric transmembrane proteins that link the cytoskeletal network within cells to ECM ligands^2^,^10^. Binding of integrins to matrix components like fibronectin^11^ leads to the recruitment of adapter proteins like Talin^12^,^13^,^14^ and signaling molecules like focal adhesion kinase (FAK)^15^,^16^. Since FAs are dynamic structures that exhibit a high degree of plasticity, FA formation is closely coupled to FA disassembly. FA dissolution is facilitated by microtubules and the calcium-dependent protease m-calpain^17^,^18^,^19^.

Altered lipid signaling has been identified as a fundamental aspect of FA dynamics. One of the most prominent lipid derivatives present at FAs is phosphatidylinositol 4,5-bisphosphate (PI4,5P_2_)^20^; and generation of this lipid seems to be essential for both formation and disassembly of FAs. Site specific generation of PI4,5P_2_ in cells is mediated by phosphatidylinositol-4-phosphate 5-kinase type-l gamma (PIP5Klγ) enzymes, using phosphatidylinositol-4-phosphate (PI4P) as substrate^21^. Multiple splice variants of PIP5Klγ have been identified^22^, and isoform 2; PIP5Klγ_i2; PIP5Klγ90; PIP5Klγ668 (referred to as PIP5Klγ in this text) is specifically targeted to the FAs^23^,^24^. Depletion of this isoform of PIP5Klγ leads to severe attachment and cytoskeletal defects in cells^25^. At the FAs, PIP5Klγ binds the Talin FERM domain, an interaction which enhances its lipid kinase activity^23^. Alterations in PI4,5P_2_ levels are necessary for proper FA function and have been implicated in regulating FA dynamics. PI4,5P_2_ formation has been shown to link integrin signaling to FAK activation^26^. Moreover, PI4,5P_2_ binding is required for oligomerization of vinculin and the control of vinculin dynamics and turnover in FAs^27^.

The dynamic regulation of FAs is intimately linked to actin cytoskeletal reorganization events^3^. The serine-threonine kinase, Protein Kinase D1 (PKD1), has been implicated in multiple biological processes including regulation of epithelial to mesenchymal transition (EMT), proliferation and regulation of actin cytoskeletal reorganization^28^,^29^,^30^. Downstream of active RhoA, PKD1 regulates actin remodeling at multiple levels. For example, PKD1 phosphorylates the phosphatase slingshot 1 L (SSH1L) and p21-activated kinase 4 (PAK4) attenuating actin remodeling processes and effectively blocking cell migration by inhibiting cofilin^31^,^32^,^33^,^34^. In addition, PKD1 was shown to regulate cell migration by stimulating the delivery of αvβ3 integrin to nascent FAs^35^, and PKD1-dependent phosphorylation of vasodilator-stimulated phosphoprotein (VASP) has been shown to drive VASP from FAs to the lamellipodium^36^. The results from the aforementioned studies indicate that PKD1 could be an integral component of the signaling nexus that controls FA function.

In the present study, we show that in response to fibronectin-mediated integrin engagement, PKD1 can be localized to the FAs where it regulates adhesion turnover and maturation downstream of RhoA. We also show that PKD1 function at the FAs is mediated through phosphorylation of FA-localized PIP5Klγ at a previously undescribed phosphorylation site, serine residue 448. The outcome of this phosphorylation is a decrease in PIP5Klγs’ lipid kinase activity and binding affinity for Talin, with the net effect of enhanced cell adhesion.

## Results

### A subcellular pool of PKD1 localizes to focal adhesions where it decreases focal adhesion turnover and increases maturation

**Figure 1:**
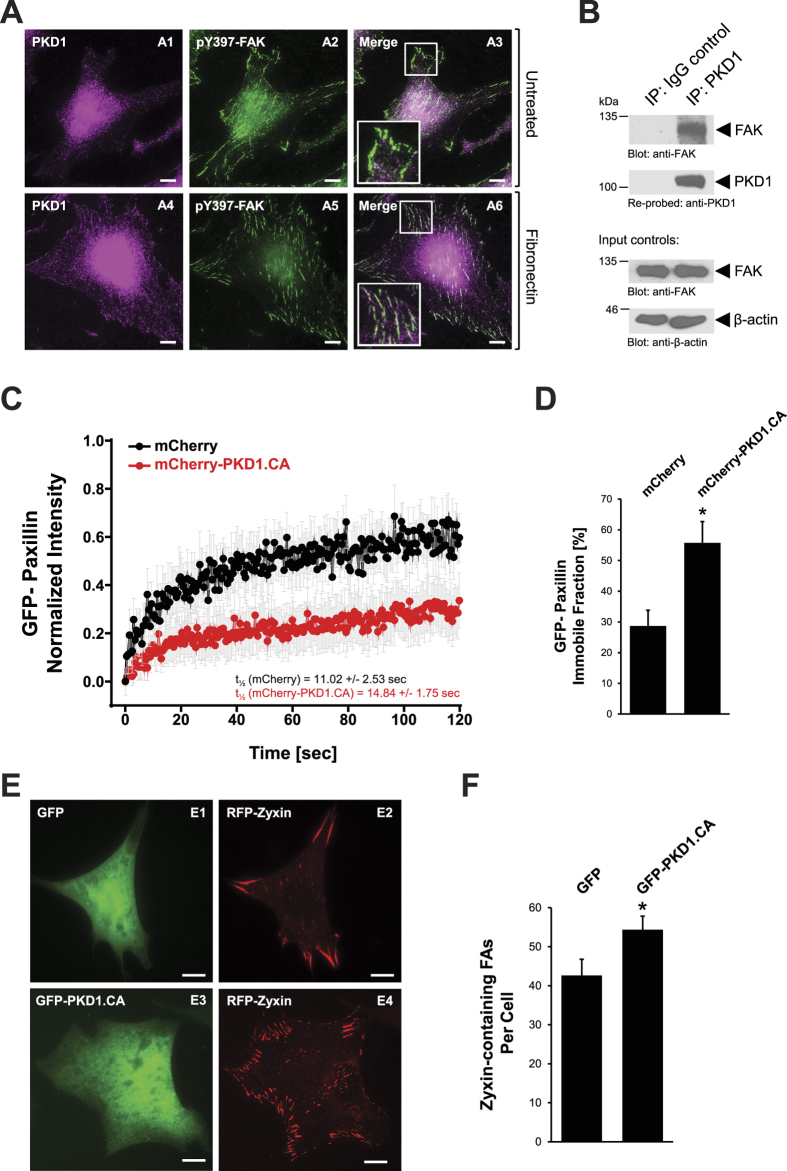
A subcellular pool of PKD1 localizes to focal adhesions where it decreases focal adhesion turnover and increases maturation. (**A**) NIH-3T3 cells (0.05 × 10^6 ^cells/well, 24 well plate) were seeded on fibronectin-coated (2 μg/ml) glass coverslips and serum-starved for 16 hrs. Cells were then simulated with 10 μg/ml fibronectin or left untreated. After 30 minutes, cells were fixed and the localization of endogenous PKD1 and pY397-FAK was determined by immunofluorescence analysis. Scale bars indicate 10 μm. Insets in A3 and A6 are 2-fold enhanced. (**B**) NIH-3T3 cells (0.8 × 10^6 ^cells/6 cm dish) were serum-starved for 16 hours, and then stimulated with 10 μg/ml fibronectin for 30 minutes. Endogenous PKD1 was immunoprecipitated and analyzed for co- immunoprecipitated FAK. Samples were re-probed for PKD1, and control Western blots of lysates were probed for FAK and β-actin. (**C**,**D**) NIH-3T3 cells were co-transfected with GFP-Paxillin and mCherry or constitutively-active mCherry-PKD1 (mCherry-PKD1.CA). FRAP analysis was conducted to determine Paxillin recovery at FAs. 3–4 focal adhesion sites per cell in at least 12 cells were analyzed for each condition. Error bars represent standard error of the mean (SEM). *Indicates statistical significance (*p* < 0.05) as compared to mCherry. (**E**) NIH-3T3 cells (0.05 × 10^6 ^cells/well, 24 well plate) expressing RFP-Zyxin and GFP or GFP-tagged constitutively-active PKD1 (GFP-PKD1.CA) were seeded on fibronectin-coated (2 μg/ml) glass coverslips. Following fixation, immunofluorescence analysis was used to determine the localization of proteins. Scale bars indicate 10 μm. (**F**) Shows quantification for the number of RFP-Zyxin positive adhesions at the periphery of cells expressing GFP (n = 33) or GFP-PKD1.CA (n = 55). *Indicates statistical significance (*p* < 0.05) as compared to control cells expressing GFP. All experiments have been performed at least three times with similar results.

Given that PKD1 is a well-established regulator of cell migration that can localize to actin-rich structures^31^,^32^,^33^, we sought to determine if PKD1 could localize to other sites in cells involved in directed cell migration. We found that after engagement of integrins by stimulation with fibronectin, endogenous PKD1 is partially co-localized with pY397-phosphorylated (catalytic-active) FAK (Fig. 1A6). In the absence of fibronectin stimulation, PKD1 co-localization with activated FAK was not observed (Fig. 1A3). To ensure that localization of PKD1 at the FAs was not antibody or cell line specific, we conducted immunofluorescence analysis with a different PKD1 antibody and in a different cell line, and a similar co-localization between PKD1 and pY397-FAK was observed (Supplemental Figure S1A,B). We next tested if PKD1 can interact directly with FAK. Therefore, in cells stimulated with fibronectin, we performed a co-immunoprecipitation in which we immunoprecipitated endogenous PKD1 and probed for endogenous FAK. In fibronectin stimulated cells, FAK can interact with PKD1 (Fig. 1B). Additionally, we found that ectopically-expressed FAK can associate with both wild type and constitutively-active PKD1 (Supplemental Figure S1C,D).

To further define the role of PKD1 at FAs we first conducted fluorescence recovery after photobleaching (FRAP) analysis to determine focal adhesion turnover. Therefore, we evaluated the recovery of GFP-Paxillin, an FA-localized scaffold protein, after expressing mCherry-PKD1.CA or mCherry as a control (Fig. 1C). While we found no significant difference in the halftime of maximum recovery of paxillin in cells expressing mCherry (t_1/2_ = 11.02 +/− 2/.53) or mCherry-PKD1.CA (t_1/2_ = 14.84 +/− 1.75), we found that the immobile fraction of GFP-paxillin was almost doubled in cells expressing mCherry-PKD1.CA as compared to control cells (Fig. 1C,D). The reduced turnover of GFP-Paxillin at the FAs suggested that PKD1 contributes to the stability of focal adhesion sites. To test this we evaluated the effect of active PKD1 on the distribution of Zyxin, an FA-localized protein that is enriched in mature adhesions^37^. In presence of active PKD1, we noticed a significant increase in the number of Zyxin-containing adhesions at the periphery of cells (Fig. 1E (compare E2 with E4) and Fig. 1F). Collectively, the results from the aforementioned experiments demonstrate that PKD1 can localize to the FAs where it affects the dynamics of FAs by promoting adhesion stabilization and maturation.

### The focal adhesion molecule PIP5KIγ is a PKD1 substrate and is phosphorylated at serine residue 448

**Figure 2:**
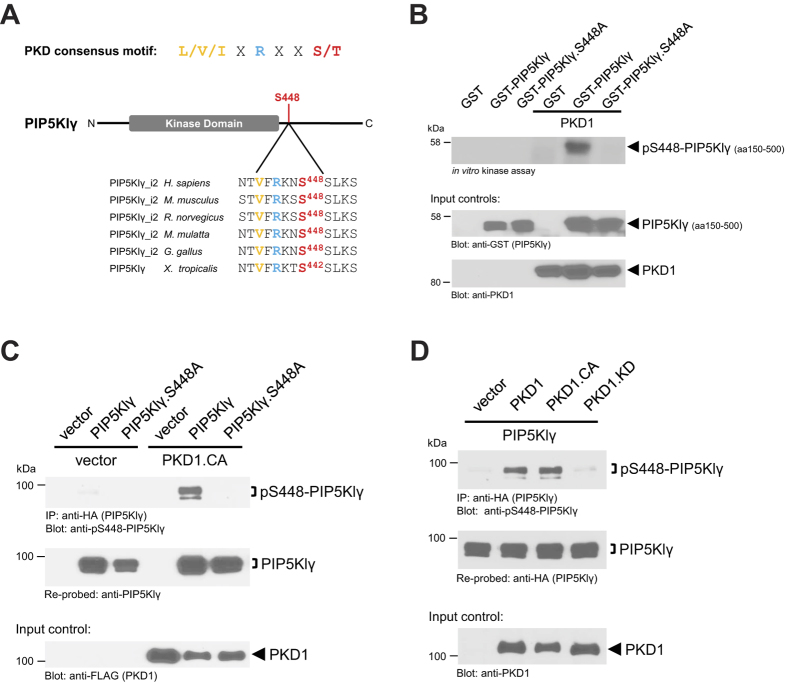
The focal adhesion molecule PIP5KIγ is a PKD1 substrate and is phosphorylated at serine residue 448. (**A**) Illustrated is the PKD consensus phosphorylation motif along with the potential PKD phosphorylation site at serine 448 in human PIP5Klγ and various other species. (**B**) An *in vitro* kinase assay was conducted using bacterially-expressed and purified GST (negative control), GST-PIP5Klγ or GST-PIP5Klγ.S448A with purified active PKD1. PKD1-mediated phosphorylation of PIP5Klγ was determined by autoradiography. Control blots show equal substrate loading of PIP5Klγ and PIP5Klγ.S448A (anti-GST) and equal input of PKD1 (anti-PKD1). (**C**) Hek293T cells (0.5 × 10^6 ^cells/well, 6 well plate) were co-transfected with vector control or FLAG-tagged constitutively-active PKD1 (PKD1.CA) and vector, HA-tagged PIP5Klγ or PIP5Klγ.S448A, as indicated. PIP5Klγ was immunoprecipitated (anti-HA) and assessed for PKD1-mediated phosphorylation using a pS448-PIP5Klγ antibody. Immunoprecipitates were re-probed with anti-PIP5Klγ for equal loading of PIP5Klγ and PIP5Klγ.S448A. The lysates were evaluated for expression of PKD1 (anti-FLAG). (**D**) Hek293T cells (0.5 × 10^6 ^cells/well, 6 well plate) were co-transfected with HA-tagged PIP5Klγ along with vector control or FLAG-tagged versions of PKD1, constitutively-active PKD1 (PKD1.CA) or kinase-dead PKD1 (PKD1.KD). An anti-HA antibody was used to immunoprecipitate PIP5Klγ from cells. Immunoprecipitates were subjected to SDS PAGE, and assessed using a pS448-PIP5Klγ antibody. Samples were counterstained for total PIP5Klγ using an anti-HA antibody. Control blots were performed for PKD1. All experiments have been performed at least three times with similar results.

PKD1 mediates many of its biological functions by phosphorylating substrates present at distinct cellular compartments^31^,^36^,^38^,^39^. Since we have shown that FA dynamics can be regulated by PKD1, we next aimed on identifying potential substrates of PKD1 that are specifically localized at the FAs, such as PIP5Klγ (Supplemental Figure S2A). An *in silico* screen using the PKD consensus phosphorylation motif^40^ identified serine residue 448 in PIP5Klγ as a potential PKD1 phosphorylation site (Fig. 2A). This site is conserved between species from *H*. *sapiens* to *X*. *tropicalis*. To determine if PIP5Klγ is actually a PKD1 substrate, we performed an *in vitro* kinase assay using bacterially-expressed GST-tagged truncated (aa150–500) versions of PIP5Klγ or PIP5Klγ.S448A in combination with recombinant active PKD1. PKD1 phosphorylated PIP5Klγ, while a PIP5Klγ.S448A mutant was not phosphorylated (Fig. 2B).

Next, we determined if PKD1-mediated phosphorylation of PIP5Klγ at this residue occurs in cells. Therefore, we co-expressed vector control, full-length PIP5Klγ or PIP5Klγ.S448A with active PKD1. In the first instance, immunoprecipitates were probed with an antibody that recognizes PKD1 phosphorylated substrates (pMOTIF antibody). Our results were consistent with the *in vitro* kinase assay, indicating that PKD1 phosphorylates PIP5Klγ and that this phosphorylation is inhibited when serine 448 is mutated to an alanine (Supplemental Figure S2B). This experiment also indicates that there is only one PKD1 phosphorylation site present in PIP5Klγ. Next we generated a phospho-site specific antibody directed against phospho-S448-PIP5Klγ. Use of this antibody in a similar experiment as described above confirmed that PKD1 indeed phosphorylates PIP5Klγ at S448 (Fig. 2C). This was also observed in another cell line, thus demonstrating that this phosphorylation event is not cell line dependent (Supplemental Figure S2C). Comparing alleles of different activation state (wildtype, constitutively-active or kinase-dead versions) indicated that PKD1 activity is needed to mediate PIP5Klγ S448 phosphorylation (Fig. 2D). In addition to this, we combined a typical PKD1 activator (PMA) with a PKD inhibitor, CRT0066101. Stimulation of cells with PMA increased PIP5Klγ S448 phosphorylation, and this was blocked in presence of CRT0066101 (Supplemental Figure S3A). Eventually, we tested if all three PKD isoforms can mediate this phosphorylation and identified PKD1 as the most potent inducer (Supplemental Figure S3B).

Taken together, these results confirm that PKD1 phosphorylates the FA-localized protein PIP5Klγ at serine 448 both *in vitro* and *in vivo*.

### Phosphorylation of PIP5KIγ at S448 does not alter its localization, but decreases activity and binding to Talin

**Figure 3:**
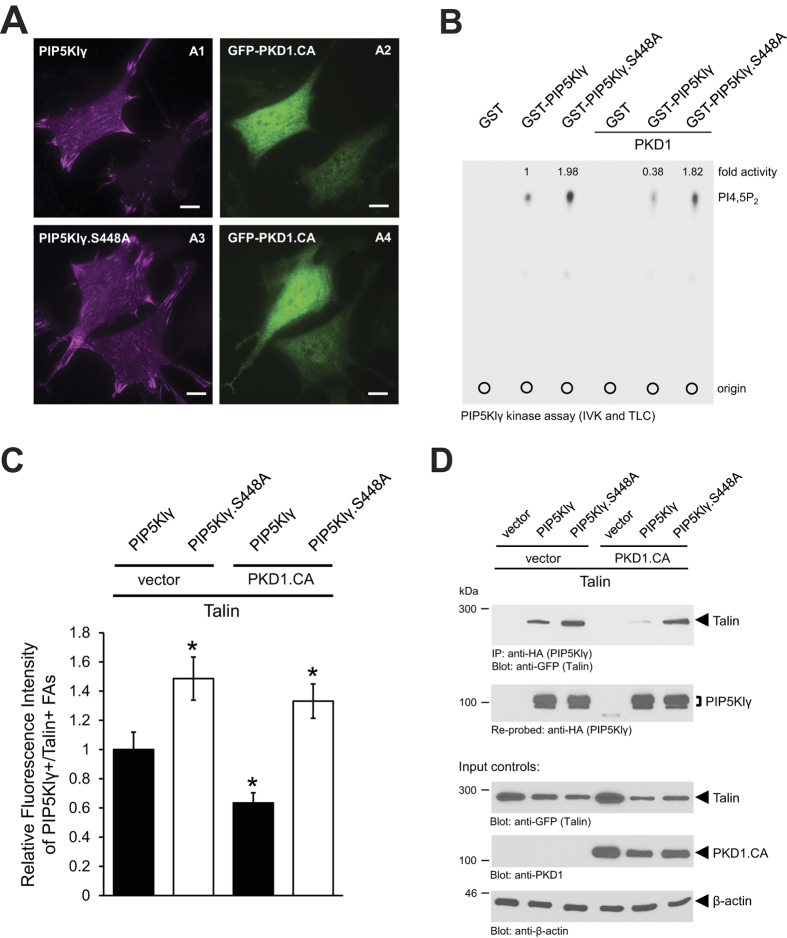
Phosphorylation of PIP5KIγ at S448 does not alter its localization, but decreases activity and binding to Talin. (**A**) NIH-3T3 cells co-expressing HA-tagged PIP5Klγ or PIP5Klγ.S448A and GFP-tagged active PKD1 (GFP-PKD1.CA) were seeded on fibronectin-coated (2 μg/ml) glass coverslips (0.05 × 10^6 ^cells/coverslip). Following fixation, immunofluorescence analysis was used to determine the localization of PKD1 and PIP5Klγ. Scale bars indicate 10 μm. (**B**) An *in vitro* lipid kinase assay was conducted using bacterially-expressed and purified GST (negative control), GST-PIP5KIγ or GST-PIP5KIγ.S448A in presence of purified PI(4)P and purified active PKD1, as indicated. Following the kinase reaction, samples were separated on a thin layer chromatogram and PIP5KIγ-mediated PI4,5P_2_ production was determined by autoradiography. (**C**) Shows quantification of the fluorescence intensity of Talin^+^/PIP5Klγ^+^ focal adhesions (n = 40 cells; n = 400 FA in total). Analysis was conducted using Image J software. *Indicates statistical significance (*p* < 0.05), as compared to vector/Talin/PIP5Klγ transfected cells (first bar). (**D**) Hek293T cells (0.5 × 10^6 ^cells/well, 6 well plate) were co-transfected with GFP-tagged Talin, vector control or FLAG-tagged active PKD1 (PKD1.CA) and vector control, HA-tagged PIP5Klγ or PIP5Klγ.S448A, as indicated. PIP5Klγ was immunoprecipitated (anti-HA) and analyzed for co-precipitated Talin (anti-GFP). Immunoprecipitates were then re-probed for PIP5Klγ (anti-HA). Control Western blots were probed for Talin (anti-GFP), PKD1 and β-actin. All experiments have been performed at least three times with similar results.

Next, we determined the functional consequences of PIP5Klγ phosphorylation by PKD1. First, we tested if PIP5Klγ localization is affected in absence or presence of constitutively-active PKD1. We found that both wild type PIP5Klγ and mutant PIP5Klγ.S448A were localized to FAs either in absence or presence of constitutively-active PKD1 (Fig. 3A and Supplemental Figure S4A for additional controls). There also was no significant difference in the distribution pattern of F-actin in cells expressing either PIP5Klγ or PIP5Klγ.S448A (Supplemental Figure S4B).

Since serine 448 lies adjacent to the kinase domain of PIP5Klγ (Fig. 2A), we tested if its lipid kinase activity was affected by serine 448 phosphorylation. An *in vitro* lipid kinase assay showed that compared to wild type PIP5Klγ, PIP5Klγ.S448A was twice as efficient in producing PI4,5P_2_. Furthermore, while the introduction of active PKD1 attenuated PIP5Klγ-mediated PI4,5P_2_ production by about 60%, the PI4,5P_2_ -producing capacity of the mutant PIP5Klγ.S448A remained largely unaffected in the presence of PKD1 (Fig. 3B). It was shown that interaction with Talin enhances PIP5Klγs’ lipid kinase activity^23^. Therefore, we next evaluated the effect of S448 phosphorylation on the interaction between these molecules.

First, we performed an immunofluorescence analysis in which we determined the localization of GFP-Talin and PIP5Klγ or PIP5Klγ.S448A in absence or presence of constitutively-active PKD1 (mCherry-PKD1.CA) (Supplemental Figure S5). Due to increase lipid kinase activity, co-localization between PIP5Klγ.S448A and Talin was much more significant than the co-localization between PIP5Klγ and Talin (Supplemental Figure S5, compare A3 with B3). Furthermore, in presence of an active PKD1, the co-localization between wildtype PIP5Klγ and Talin diminished considerably (Supplemental Figure S5, compare A3 with C3), while the co-localization between PIP5Klγ.S448A and Talin remained unaffected (Supplemental Figure S5, compare B3 with D3). Quantification of fluorescence intensities of Talin^+^/PIP5Klγ^+^ FAs revealed that the combined intensity of Talin and PIP5Klγ.S448A was about 50% more than the combined fluorescence intensity of Talin and PIP5Klγ. There was a robust decrease in the combined fluorescence intensity of PIP5Klγ and Talin in the presence of active PKD1; however, no significant changes in the combined fluorescence intensity of PIP5Klγ.S448A and Talin were observed in the presence of active PKD1 (Fig. 3C).

To confirm these observations we then conducted co-immunoprecipitation assays and found that compared to wild type PIP5Klγ, mutant PIP5Klγ.S448A has a higher binding affinity for Talin (Fig. 3D, compare lanes 2 and 3). Moreover, while the binding affinity of wild type PIP5Klγ for Talin was almost completely inhibited in the presence of active PKD1 (Fig. 3D, compare lanes 2 and 5), the interaction between PIP5Klγ.S448A and Talin remained the same whether or not active PKD1 was present (Fig. 3D, compare lanes 3 and 6).

In sum, these results indicate that while phosphorylation of PIP5Klγ at serine 448 does not affect its localization, it inhibits lipid kinase activity, which then relays to a loss of interaction between PIP5Klγ and Talin.

### Phosphorylation of PIP5KIγ at S448 is regulated through a fibronectin-RhoA-PKD1 signaling pathway

**Figure 4:**
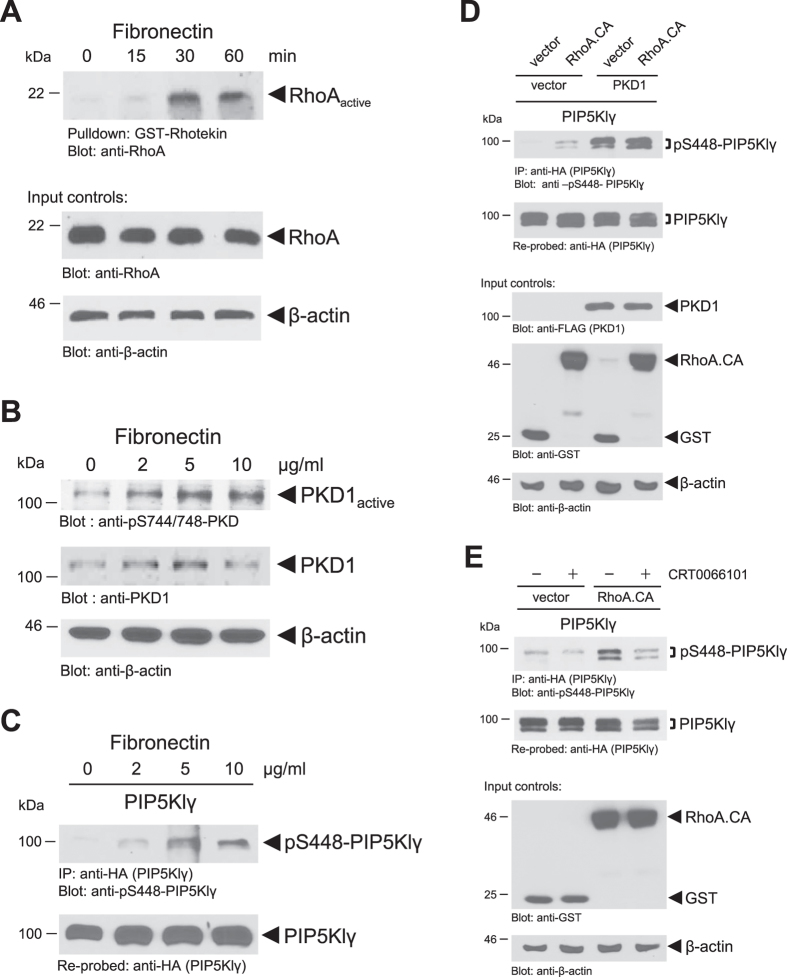
Phosphorylation of PIP5KIγ at S448 is regulated through a fibronectin-RhoA-PKD1 signaling pathway. (**A**) NIH-3T3 cells (0.5 × 10^6 ^cells/10 cm dish) were serum-starved for 24 hours. Cells were stimulated with 10 μg/ml fibronectin for the times indicated, and endogenous active RhoA was isolated from cells using a Rhotekin pulldown assay. Control blots of cell lysates were probed for total RhoA and β-actin expression. (**B**) Hek293T cells (0.6 × 10^6 ^cells/well, 6 well plate) were serum-starved overnight. Then cells were stimulated for 30 minutes with varying concentrations of fibronectin as indicated. Cell lysates were evaluated for PKD1 activity with the use of a pS744/748-PKD antibody and re-probed for PKD1 (anti-PKD1). Control blots were probed for β-actin. (**C**) Hek293T cells (0.6 × 10^6 ^cells/well, 6 well plate) were transfected with HA-tagged PIP5Klγ and serum-starved overnight. Then cells were stimulated for 30 minutes with varying concentrations of fibronectin as indicated. PIP5Klγ was immunoprecipitated (anti-HA) and analyzed using a pS448-PIP5Klγ antibody. Samples were re-probed with anti-HA to determine PIP5Klγ loading. (**D**) Hek293T cells (0.5 × 10^6 ^cells/well, 6 well plate) were co-transfected with HA-tagged PIP5Klγ, vector or FLAG-tagged PKD1 and vector or GST-tagged active RhoA (RhoA.CA), as indicated. PIP5Klγ was immunoprecipitated (anti-HA) and analyzed using pS448-PIP5Klγ antibody. Samples were re-probed with anti-HA to determine PIP5Klγ loading. Control Western blots were probed for PKD1 (anti-FLAG), RhoA.CA (anti-GST) and β-actin expression. (**E**) Hek293T cells (0.5 × 10^6 ^cells/well, 6 well plate) were co-transfected with HA-tagged PIP5Klγ and vector control or GST-tagged active RhoA (RhoA.CA). Cells were stimulated with CRT0066101 (2.5 μM, 1 hour) or left untreated as indicated. PIP5Klγ was immunoprecipitated (anti-HA) and analyzed using the pS448- PIP5Klγ antibody. Immunoprecipitates were re-probed for total PIP5Klγ (anti-HA). Control blots were probed for RhoA.CA (anti-GST) and β-actin expression. All experiments have been performed at least three times with similar results.

Next, we explored the upstream signaling leading to PKD1-mediated phosphorylation of PIP5Klγ. A typical activator of PKD1 that also has been linked to focal adhesion kinetics is the RhoGTPase RhoA^32^,^36^. Since endogenous PKD1 localized to the FA after integrin engagement with fibronectin (Fig. 1A), we first assessed the ability of fibronectin to induce RhoA activation using a Rhotekin pulldown assay. We found that RhoA activation occurred after 15 minutes and was maximally activated after 30 minute stimulation with fibronectin (Fig. 4A). Treatment of cells with increasing concentrations of fibronectin for 30 minutes induced the activation of endogenous PKD1, as judged by monitoring its phosphorylation at the activation loop serines using an anti-pS744/748 antibody (Fig. 4B). Moreover, PKD1 activity directly correlated with phosphorylation of PIP5Klγ at S448 (Fig. 4C).

Since fibronectin activates RhoA as well as PKD1 (Fig. 4A,B), and RhoA previously has been recognized as an activator for PKD1^32^,^36^, we then tested if RhoA-mediated activation of PKD1 leads to phosphorylation of PIP5Klγ at S448. Expression of an active allele of RhoA or of PKD1 alone induced phosphorylation of PIP5Klγ at S448 and this was further increased when both proteins were present (Fig. 4D). Eventually, we found that the robust phosphorylation of PIP5Klγ at S448 induced by a constitutively-active version of RhoA is almost completely blocked when PKD is inhibited (Fig. 4E), demonstrating that PKD1 indeed operates downstream of RhoA to phosphorylate PIP5Klγ. Once more, we demonstrated that PKD1-mediated phosphorylation of S448 downstream of fibronectin-RhoA signaling is not limited to a single cell type (Supplemental Figure S6A,B).

In summary, these results suggest that S448 phosphorylation of PIP5Klγ is mediated by a fibronectin-RhoA-PKD1 signaling cascade.

### PKD1 mediated phosphorylation of PIP5KIγ regulates cell adhesion

**Figure 5:**
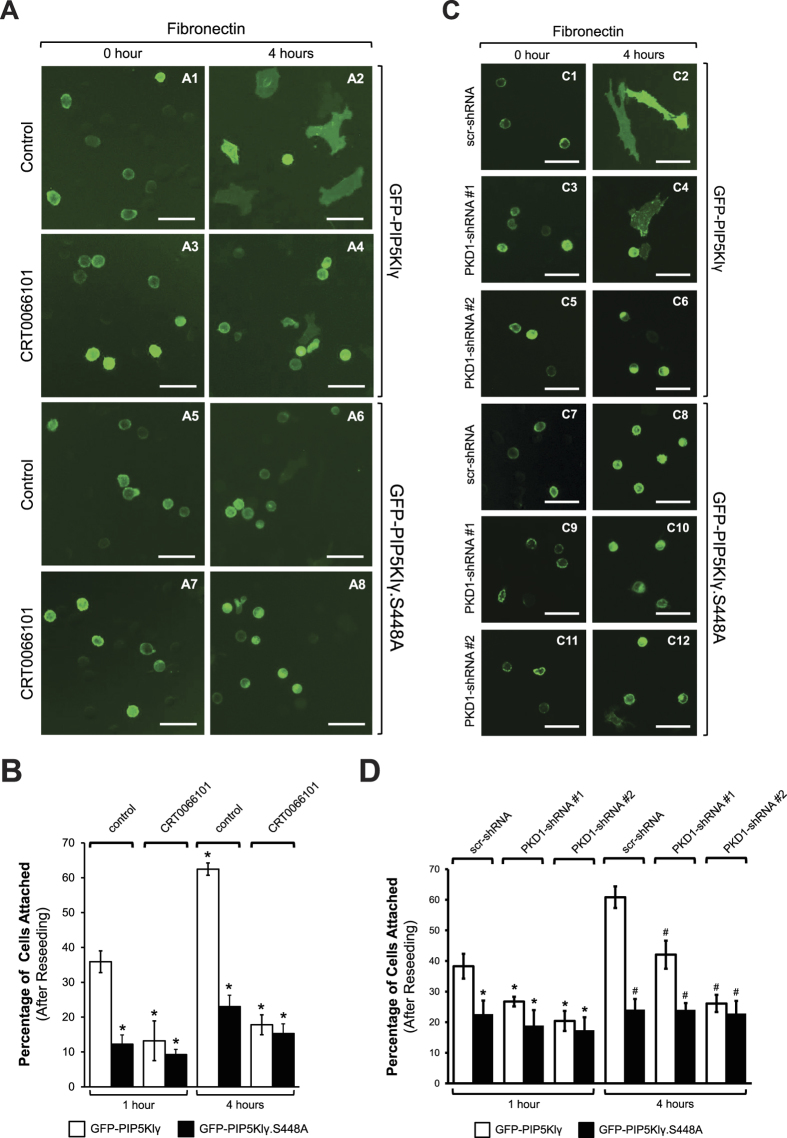
PKD1 mediated phosphorylation of PIP5KIγ regulates cell adhesion. (**A**) NIH-3T3 cells (0.4 × 10^6 ^cells/well, 6 well plate) expressing GFP-PIP5Klγ or GFP-PIP5Klγ.S448A were treated with CRT0066101 (10 μM) or left untreated. After 60 minutes, the cells were detached and re-plated on fibronectin-coated (10 μg/ml) glass coverslips (0.05 × 10^6 ^cells/coverslip). Fluorescent images were acquired at the indicated times to determine the number of cells attached. Bars indicate 50 μm. (**B**) Shows quantification for the percentage of cells attached at 1 and 4 hours. Error bars shown represent (SEM) and represent three independent experiments. *Indicates statistical significance (*p* < 0.05) as compared to untreated cells expressing GFP-PIP5Klγ 1 hour after re-plating. (**C**) NIH-3T3 cells (0.3 × 10^6 ^cells/well, 6 well plate) were infected with lentivirus expressing non-target (scr) shRNA, or two independent shRNA sequences targeting PKD1. After 48 hours, cells were transfected with GFP-PIP5Klγ or GFP-PIP5Klγ.S448A, and 16 hours post transfection, cells were detached and re-plated on fibronectin-coated (10 μg/ml) glass coverslips (0.05 × 10^6 ^cells/coverslip). Fluorescent images were acquired at the indicated times to determine the number of cells attached. Bars indicate 50 μm. (**D**) Shows quantification for the percentage of cells attached at 1 and 4 hours. Error bars shown represent (SEM) and represent three independent experiments. *Indicates statistical significance (*p* < 0.05) as compared to cells expressing scr-shRNA and GFP-PIP5Klγ 1 hour after re-plating. ^#^Indicates statistical significance (*p* < 0.05) as compared to cells expressing scr-shRNA and GFP-PIP5Klγ 4 hours after re-plating.

Since integrin engagement facilitates cellular attachment, we next determined if the stabilization of focal adhesions by PKD1-PIP5Klγ signaling affects cell adhesion. The attachment of NIH-3T3 fibroblasts to fibronectin usually occurs within 30 minutes (Supplemental Figure S7A). When comparing adhesion of cells that express either wildtype or mutant PIP5Klγ to fibronectin, we observed that the PIP5Klγ.S448A mutant showed a significant decrease in cell adhesion (Fig. 5A, compare A2 and A6). To determine a role for PKD1-PIP5Klγ signaling in the cell adhesion process, we additionally treated cells with CRT0066101. Inhibition of PKD with CRT0066101 decreased the ability of cells expressing wild type PIP5Klγ to attach to fibronectin to a level as observed with PIP5Klγ.S448A (Fig. 5A, compare A2 with A4 and A6). Quantification analyses at different time points, indicated that 1 hour after reseeding approximately 30% of the cells expressing wild type PIP5Klγ had attached to fibronectin, but when treated with CRT0066101 or a PIP5Klγ.S448A mutant was expressed this was decreased to 10% (Fig. 5B). Similar observations were made after 4 hours with approximately 60% of cells attached that express wild type PIP5Klγ, and reduction of attached cells to approximately 20% after treatment with CRT0066101 or expression of PIP5Klγ.S448A (Fig. 5B).

To confirm that the adhesion defects seen in PIP5Klγ-expressing cells in response to stimulation with CRT0066101, was actually mediated specially by a loss of PKD1 kinase activity, we utilized a reverse genetics approach. We evaluated the adhesion capacity of PIP5Klγ and PIP5Klγ.S448A-expressing cells harboring non-target scr-shRNA or two distinct shRNA sequences targeting PKD1 (Western blots shown in Supplemental Fig. S7B). Scr-shRNA infected cells expressing PIP5Klγ were more adherent than cells expressing PIP5Klγ.S448A (Fig. 5C, compare C2 with C8). PKD1-shRNA in PIP5Klγ-expressing cells diminished their ability to attach to fibronectin (Fig. 5C, compare C2 with C4 and C8). Quantification analyses (Fig. 5D) showed that 40% of control cells were attached 1 hour after reseeding and 60% after 4 hours, whereas the knockdown of PKD1 decreased attachment at 1 hour to about 25% (shRNA #1) and 20% (shRNA #2) and at 4 hours to 40% (shRNA #1) and 25% (shRNA #2). PIP5Klγ.S448A-expressing cells generally had difficulties to attach and the number of cells attached 1 or 4 hours after reseeding remained largely unaffected by the expression level of PKD1 (Fig. 5D).

Our results indicate that ectopic expression of a PIP5Klγ mutant (PIP5Klγ.S448A) that shows increase lipid kinase activity significantly decreases cell adhesion. Ectopic expression of wild type PIP5Klγ as compared to parent cells also showed some delay in adhesion, but this was not as pronounced as observed with the mutant. Moreover, phosphorylation of PIP5Klγ by PKD1 (and subsequent decrease in lipid kinase activity) is an important requirement for the adhesion process.

## Discussion

Formation of focal adhesions is initiated by the binding of integrins to ECM ligands and the subsequent recruitment of proteins like Talin and FAK^2^,^14^,^15^,^41^. Given the dynamic nature of FA function and structure, FA assembly is closely associated with FA disassembly^17^,^18^.

The serine/threonine kinase, PKD1, has been shown to impact various biological processes related to cell-cell contacts, EMT and motility^28^,^32^,^42^,^43^,^44^,^45^. With respect to its role in FA formation, it was shown that PKD1 promotes αvβ3 integrin recycling and delivery to nascent focal adhesions during cell spreading^35^. Otherwise, there is no information on how PKD1 can affect focal adhesion dynamics. We show that in response to fibronectin-mediated engagement of integrins, endogenous PKD1 can localize to the FAs where it co-localizes with activated FAK (Fig. 1A). Co-immunoprecipitation analyses show that PKD1 and FAK are part of the same protein complex, an interaction also likely mediated by fibronectin-mediated engagement of integrins (Fig. 1B). However, it needs to be noted that PKD1 within cells also can localize to the cytosol, nucleus, golgi, plasma membrane and mitochondria^29^. Due to this ubiquitous localization (as well as high expression levels) the presence of ectopically-expressed fluorescent protein-tagged PKD1 at FAs was difficult to see in immunofluorescence, although it can be co-immunoprecipitated with FAK and functionally affected the FAs. Introduction of an active PKD1 into cells inhibited the turnover of Paxillin localized at FAs and increased the number of Zyxin-positive FAs (Fig. 1). Both suggest that PKD1 affects the dynamics of FAs by decreasing turnover and enhancing maturation. Since directed cell migration is dependent on both formation and dissolution of FAs^5^, PKD1-mediated FA stabilization is in accord with the multiple studies showing that active PKD1 can block cell migration and invasion^31^,^32^,^34^,^36^,^46^.

With PIP5Klγ_i2, the FA-localized isoform of PIP5Klγ^23^,^24^, we now identify a new PKD1 target whose phosphorylation regulates FA dynamics and cell adhesion. With serine 448 we mapped a new PKD1-regulated phosphorylation site in this lipid kinase (Fig. 2), which is regulated by a Fibronectin-RhoA-PKD1 signaling pathway (Fig. 4 and Supplemental Figure S6). Phosphorylation by PKD1 did not affect the localization of PIP5Klγ (Fig. 3A and Supplemental Figure S4), which is not surprising since localization of PIP5Klγ to FAs is dependent on its C-terminal amino acids^23^,^24^. Instead, it inhibited its lipid kinase activity and binding to Talin (Fig. 3B–D and Supplemental Figure S5), which both can negatively-affect FA turnover. Previous work has shown that interaction of PIP5Klγ with Talin increases its lipid kinase activity^23^. An interaction between both molecules is promoted when PIP5Klγ is phosphorylated at Y649^47^, and is blocked after phosphorylation at S650^48^. However, other phosphorylation events have been described to block this interaction, including phosphorylation at S555 by Akt^49^, or as described in the present study, phosphorylation at S448 by PKD1. The simplest explanation for this is that Talin binds to PIP5Klγ through multiple residues. Another possibility is that some of these phosphorylations induce conformational changes in PIP5Klγ that either affect binding to protein partners or affect accessibilities of phosphorylation sites to upstream kinases.

PI4,5P_2_, generated by PIP5Klγ at the FAs, has emerged as a critical regulator of FA dynamics^23^,^24^. PI4,5P_2_ promotes FA formation by binding and activating many FA-localized proteins^27^,^50^, but also promotes FA disassembly^51^. Such dual roles in the turnover of FAs for PI4,5P_2_ may be explained by a local imbalance in PI4,5P_2_ homeostasis^23^. In our hands, cells overexpressing PIP5Klγ showed a delay in attachment when compared to wildtype cells (Fig. 5A,C *versus* Supplemental Figure S7A). However, adhesion to fibronectin was impaired in cells expressing PIP5Klγ.S448A, a mutant with increased lipid kinase activity (Figs 5A,C and 3B). Moreover, similar effects were obtained in cells expressing wildtype PIP5Klγ when PKD1 activity was inhibited or its expression was reduced. Inhibition of PIP5Klγ by PKD1 and subsequent decreased activity, limits the pool of PI4,5P_2_ present at FAs. As a result thereof, PI4,5P_2_-dependent processes which promote FA formation such as vinculin oligomerization^27^, as well as processes that promote FA dissolution such as m-calpain-mediated proteolysis^52^ are inhibited. Naturally, inhibition of these processes fundamental to FA turnover would result in the stabilization of FAs.

The inhibition of binding of PIP5Klγ to Talin after its phosphorylation by PKD1, in addition to effects of the lipid product PI4,5P_2_, adds another layer of regulation of FA formation and stability. It was shown that Src-mediated phosphorylation of PIP5Klγ enhances its binding affinity for Talin, and inhibits the binding of β-integrin to Talin^47^, suggesting that the interactions between Talin and PIP5Klγ and Talin and β-integrin are mutually exclusive. Consequently, the less Talin is bound to PIP5Klγ (as seen in the presence of PKD1), the more Talin is bound to β-integrin, as a stabilizing structural element of FAs^12^.

**Figure 6:**
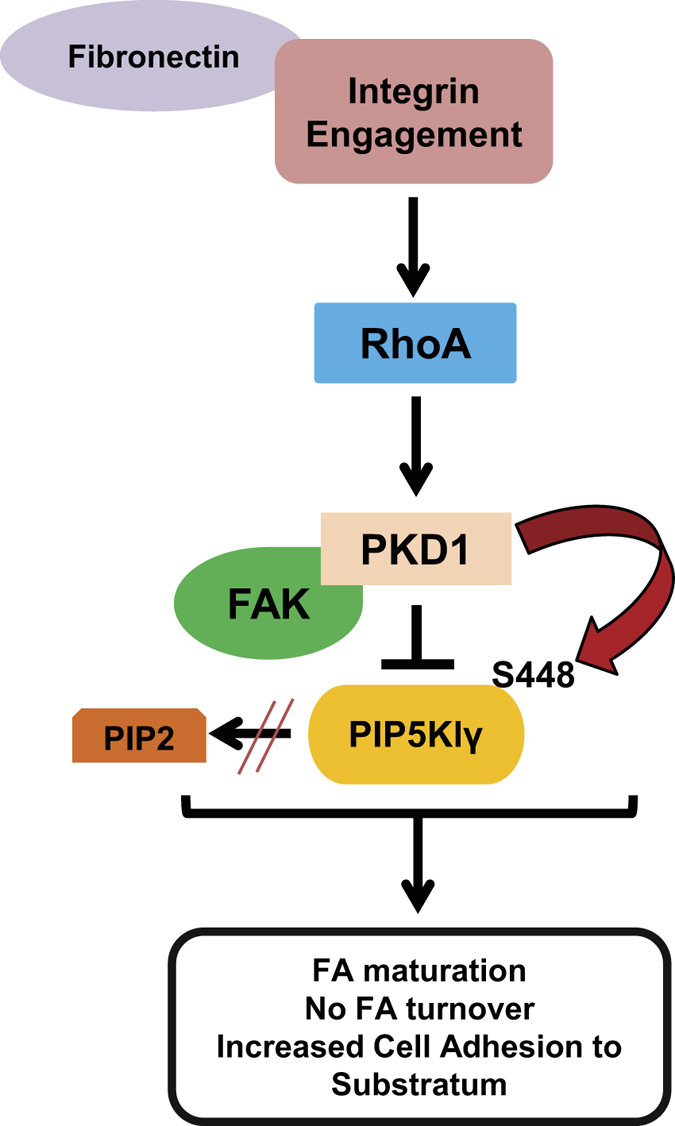
Model of how PKD1 affects focal adhesion dynamics. In cells stimulated with fibronectin, a subcellular pool of PKD1 localizes to FAs where it induces FA maturation and blocks FA turnover. This is mediated by PKD1 phosphorylation of FA-localized PIP5Klγ at serine 448 which occurs in response to Integrin-RhoA signaling. S448 phosphorylation inhibits PIP5Klγs’ binding affinity for Talin and its lipid kinase activity which in turn leads to increased cellular adhesion.

In summary, we show that integrin engagement after stimulation with fibronectin can drive a pool of PKD1 to the FAs where it negatively-regulates FA turnover. This is mediated by phosphorylation of PIP5Klγ downstream of a fibronectin-RhoA-PKD1 signaling pathway and overall promotes cell adhesion and FA stability (Fig. 6).

## Experimental Procedures

### Cell lines, Antibodies and Reagents

HeLa, Hek293T, NIH-3T3 and MCF-7 cells (from ATTC Manassas, VA) were cultured in DMEM supplemented with 10% FBS. Anti-GST, anti-GFP (B-2), anti-RhoA (119) and anti-PKD1 (C-20) antibodies were from Santa Cruz Biotechnology (Santa Cruz, CA), anti-HA, anti-FLAG (M2), and anti-β-actin from Sigma-Aldrich (St. Louis, MO). Anti-FAK was from BD Transduction Laboratories (Franklin Lakes, NJ), anti-pY397-FAK from Invitrogen (Carlsbad, CA), and anti-PIP5Klγ from Millipore (Billerica, MA). Anti-pS744/748 and anti-pMOTIF were from Cell Signaling Technology (Danvers, MA), anti-PKD1 (H00005587-A01; used for immunofluorescence shown in (Supplemental Figure S1B) was from Abnova Cooperation (Taipei City, Taiwan). The affinity-purified goat antibody specific for PKD1 (used for immunofluorescence in Fig. 1A and Supplemental Figure S1A) was generated by Everest Biotech (Oxfordshire, United Kingdom) using C-QKSPSESFIGREKR as an immunogen. The mouse monoclonal antibody against PKD1 was developed by the Storz laboratory and has been described before^53^. The affinity-purified rabbit polyclonal anti-pS448-PIP5Klγ antibody, specific for PIP5Klγ phosphorylated at serine residue 448 was developed by 21^st^ Century Biochemicals (Marlboro, MA) using two immunogens: Ac-NTVFRKN[pS]SLKSSPSK-Ahx-C-amide and C-Ahx-SNTVFRKN[pS]SLKSSPS-amide. Secondary HRP-linked antibodies were from Millipore. Secondary antibodies (Alexa Fluor 488 F(ab’)2 fragment of goat-anti-mouse IgG, Alexa Fluor 568 F(ab’)2 fragment of goat-anti-rabbit IgG & goat-anti-mouse IgG, Alexa Fluor 647 F(ab’)2 fragment of goat-anti-rat IgG & donkey-anti-goat IgG) were from Invitrogen. For transient transfection, TransIT-293 (Mirus Bio, Madison, WI) was used for Hek293T cells, TransIT-HeLa Monster (Mirus Bio) for HeLa cells and GenJet II reagent (SignaGen Laboratories, Rockville, MD) for NIH-3T3 cells. Lysozyme and deoxycholic acid were from Thermo Fisher Scientific (Waltham, MA). Fibronectin was from Sigma-Aldrich and Rhotekin-RBD beads were from Cytoskeleton Inc (Denver, CO). Phosphatidylinositol 4-phosphate diC16 [PI(4)P] was from Echelon Biosciences (Salt Lake City, UT) and CRT0066101 was from Tocris Bioscience (Bristol, UK). All other chemicals were from Thermo Fisher Scientific.

### DNA Expression and Lentiviral shRNA Expression Constructs

The expression plasmids for FLAG-tagged human PKD1, constitutively-active PKD1 (S738E/S742E), kinase-dead PKD1 (K612W), GFP-tagged constitutively-active PKD1 and pEBG-RhoA.CA have been described before^31^,^32^,^36^. The pEGFP-N1 and pmCherry-N1 vectors were from Clontech (Mountain View, CA). mCherry-PKD1 was generated by cloning full-length human PKD1 into pmCherry-N1 via Xhol and BamHl. mCherry-PKD1.CA was generated by site directed mutagenesis using QuikChange Site-Directed Mutagenesis kit (Agilent Technologies, Santa Clara, CA) and previously described primers^54^ to introduce S738E/S742E mutations. HA-tagged PIP5Klγ has been described previously^24^, RFP-Zyxin was from Dr. P. Anastasiadis (Mayo Clinic, Jacksonville, FL) and mCherry-Paxillin from Dr. K. Hu (Indiana University, Bloomington, IN). The following plasmids were obtained from Addgene: mCherry-PTK2-C-10 (plasmid #55122), GFP-Talin1 (plasmid #26724) and GFP-PIPK1 gamma 90 (plasmid #22299). A GST-tagged version of a truncated PIP5Klγ comprising amino-acids 150–500 was cloned into pGEX4-T1 via EcoRI and XhoI. PIP5KIγ S448A mutations were introduced by site directed mutagenesis using QuikChange Site-Directed Mutagenesis kit (Agilent Technologies) using 5′-ACGGTCTTTCGGAAGAACGCCTCCCTGAAGTCCTCGCCC-3′ and 5′-GGGCGAGGACTTCAGGGAGGCGTTCTTCCGAAAGACCGT-3′ as primers. The lentiviral plasmids to knockdown murine PKD1 and the control non-target (scr) shRNA plasmid were from Sigma-Aldrich and have been described in^55^.

### Pull-down Assays for Active Rho

For pull-down of active Rho, Rhotekin-Rho binding domain beads were used according to the instructions from the manufacturer (Cytoskeleton Inc.).

### *In Vitro* Kinase Assay and *In Vitro* Lipid Kinase Assay

Protein kinase assays were carried out by adding 250 ng of active, purified PKD1 enzyme to 2 μg of GST or GST-fusion protein in 40 μl of kinase buffer (50 mM Tris pH 7.4, 10 mM MgCl_2_, 2 mM DTT) supplemented with 100 μM ATP containing 10 μCi of [γ-^32^P] ATP in kinase buffer. After 30 minutes at room temperature the reaction was stopped by addition of 2× Laemmli buffer. For lipid kinase assays, active purified PKD1 was combined with GST or GST-fusion proteins and incubated for 5 minutes at room temperature in 40 μl kinase buffer (5×: 250 mM Tris pH7.5, 50 mM MgCl_2,_ 2.5 mM EDTA pH8.0), supplemented with 25 μM PI(4)P micelles and 50 μM ATP containing 1μCi of [γ-^32^P] ATP in kinase buffer. Samples were then resolved using thin layer chromatography.

### Immunoblotting, Immunoprecipitation and SDS-PAGE

Cells were washed 2 times with ice-cold PBS (140 mM NaCl, 2.7 mM KCl, 8 mM Na_2_HPO_4_, 1.5 mM KH_2_PO_4_ pH 7.2) and lysed with Buffer A (50 mM Tris-HCl pH7.4, 1% Triton X-100, 150 mM NaCl, 5 mM EDTA pH 7.4) containing Protease Inhibitor Cocktail (PIC, Sigma-Aldrich). After 30 minutes of incubation on ice, lysates were centrifuged (13,000 rpm, 15 min, 4 °C) and protein concentration was determined. Proteins of interest were immunoprecipitated by incubation with a specific antibody (2 μg) for 1 hour, after which samples were incubated for a further 30 minutes with Protein G Sepharose (GE Healthcare, Piscataway, NJ). Immunecomplexes were washed 3 times with TBS (50 mM Tris-HCl pH 7.4, 150 mM NaCl), and resolved in 20 μl TBS and 2x Laemmli buffer. Samples were subjected to SDS-PAGE, transferred to nitrocellulose membranes and visualized by immunostaining.

### Immunofluorescence

Cells were grown in 6 well plates, transfected as indicated, and reseeded onto fibronectin-coated glass coverslips (2 μg/ml) in a 24 well plate at a density of 5 × 10^4^ cells per well. At indicated time points cells were washed twice with PBS and then fixed with 4% paraformaldehyde (15 min, 37 °C). Following fixation, cells were washed two times with PBS, permeabilized with 0.1% Triton X-100 in PBS (3 min, RT) and blocked with 3% bovine serum albumin and 0.05% Tween 20 in PBS (blocking solution) for 30 min at room temperature. For the immunofluorescence in Fig. 1A, prior to blocking, cells were simultaneously fixed and permeabilized with 4% paraformaldehyde supplemented with 0.1% Triton X-100 (5 min, 37 °C) and then fixed again with 4% paraformaldehyde (15 min, 37 °C). Samples were incubated with primary antibodies (anti-PKD1 (Abnova) 1:200, anti-PKD1 (Everest Biotech) 1:500, anti-pY397-FAK 1:300, anti-HA 1:400) in blocking solution overnight at 4 °C. Following five washes with PBS, samples were incubated with secondary antibodies (Alexa Fluor 488 F(ab’)2 fragment of goat-anti-mouse IgG, Alexa Fluor 568 F(ab’)2 fragment of goat-anti-rabbit IgG, Alexa Fluor 568 F(ab’)2 fragment of goat-anti-mouse IgG, Alexa Fluor 647 F(ab’)2 fragment of donkey-anti-goat IgG and Alexa Fluor 647 F(ab’)2 fragment of goat-anti-rat IgG) diluted at 1:800 in blocking solution. After further washing in PBS, the glass coverslips were mounted onto slides using Lab Vision^TM^ PermaFluor^TM^ Aqueous Mounting Medium (Thermo Fisher Scientific) and evaluated using an IX81 DSU Spinning Disc Confocal from Olympus with a 60× objective.

### Fluorescence Recovery after Photobleaching (FRAP)

For FRAP analysis cells were transfected as indicated and seeded in 3.5 cm glass bottom culture dishes (MatTek Corporation, Ashland, MA) coated with fibronectin (2 μg/ml). Transfected cells were imaged in CEB buffer (2 mM MgCl_2_, 2 mM CaCl_2_, 150 mM NaCl, 2.5 mM KCl, 10 mM glucose and 10 mM sodium HEPES, pH 7.5) and imaged using a Zeiss LSM 510 META laser-scanning confocal microscope (Carl Zeiss Microscopy, Oberkochen, Germany) equipped with a Plan-APOCHROMAT 63× oil immersion objective (1.4 NA) and Zen (2009) software. Before FRAP was conducted, initial images were acquired with 488 nm and 594 nm excitation to confirm that cells expressed proteins of interest. For FRAP, cells were imaged with an argon/2 laser at 488 nm, and emission was collected using a band-pass 505–570IR filter with a 63× oil objective at 1.7× digital zoom. 300 images were acquired every 0.495 seconds at 256 × 256 pixel resolution, 0.33 μm × 0.33 μm pixel size and 3.21 μsec pixel dwell time. After the third image had been acquired, focal adhesions were bleached using the 405 nm diode laser (30 mW) in combination with the 458, 477 and 488 excitation wavelengths on the argon laser line (30 mW) at 100% transmission for 150 iterations (12. 79 μsec pixel dwell time during the bleach phase). Fluorescence in the bleached region and in a similarly sized and positioned non-bleached (reference) region was quantified at every time point. At least 12 cells were analyzed for each condition. FRAP curves of normalized fluorescence intensity were generated as described before^36^.

### Purification of GST Fusion Proteins

GST-fusion protein expression was induced in *E*. *coli* using 1 mM IPTG (4 hr, 37 °C). The bacterial pellets were re-suspended in suspension buffer (50 mM Tris, pH 8.0, 1 mM EDTA, 100 mM NaCl, 1 mM DTT, 100 μM PMSF, 0.02 mg/ml DNase I), lysed with 2 mg/ml lysozyme (20 min on ice) and incubated with 2 mg/ml deoxycholic acid (30 min at RT). Following sonication of the suspension, the supernatant was purified using glutathione sepharose beads (GE Healthcare). Subsequently, proteins were eluted from the beads as described previously^36^.

### Adhesions Assays

Cells were infected or transfected as indicated. In Fig. 5A, 16 hours after transfection, cells were stimulated with CRT0066101 or left untreated. One hour after treatment with CRT0066101, cells were reseeded onto fibronectin-coated glass coverslips (10 μg/ml) in a 24-well plate at a density of 5 × 10^4^ cells per well. Cells were imaged at the indicated times and counted manually to determine the number of cells attached.

### Statistical Analysis

Data are presented as mean ± SEM. P values were acquired with the student’s *t*-test using Graph Pad software, and p < 0.05 was considered statistically significant.

## Additional Information

**How to cite this article**: Durand, N. *et al*. Protein Kinase D1 regulates focal adhesion dynamics and cell adhesion through Phosphatidylinositol-4-phosphate 5-kinase type-l γ. *Sci. Rep.* **6**, 35963; doi: 10.1038/srep35963 (2016).

## Supplementary Material

Supplementary Information
