# Supplementary material for: Protein Kinase D1 regulates focal adhesion dynamics and cell adhesion through Phosphatidylinositol-4-phosphate 5-kinase type-l γ
Source: Sci Rep. 2016 Oct 24;6:35963. doi: 10.1038/srep35963 (PMC5075913; doi:10.1038/srep35963)
Supplement: Supplementary Information [file srep35963-s1.pdf]

Supplementary Information
for

Protein Kinase D1 regulates focal adhesion dynamics and cell adhesion through Phosphatidylinositol-4-phosphate 5-kinase type-I γ

Nisha Durand[1], Ligia I. Bastea[1], Jason Long[1], Heike Döppler[1], Kun Ling[2] and Peter Storz[1,3]

[1] Department of Cancer Biology, Mayo Clinic, Jacksonville, Florida 32224, USA
[2] Department of Biochemistry and Molecular Biology, Mayo Clinic, Rochester, Minnesota 55905, USA
[3] Corresponding author: Peter Storz, Mayo Clinic, 4500 San Pablo Road, Jacksonville, FL 32224. Phone: 904 953-6909, FAX: 904 953-0277, e-mail: storz.peter@mayo.edu

Supplemental Figure S1, relates to Figure 1

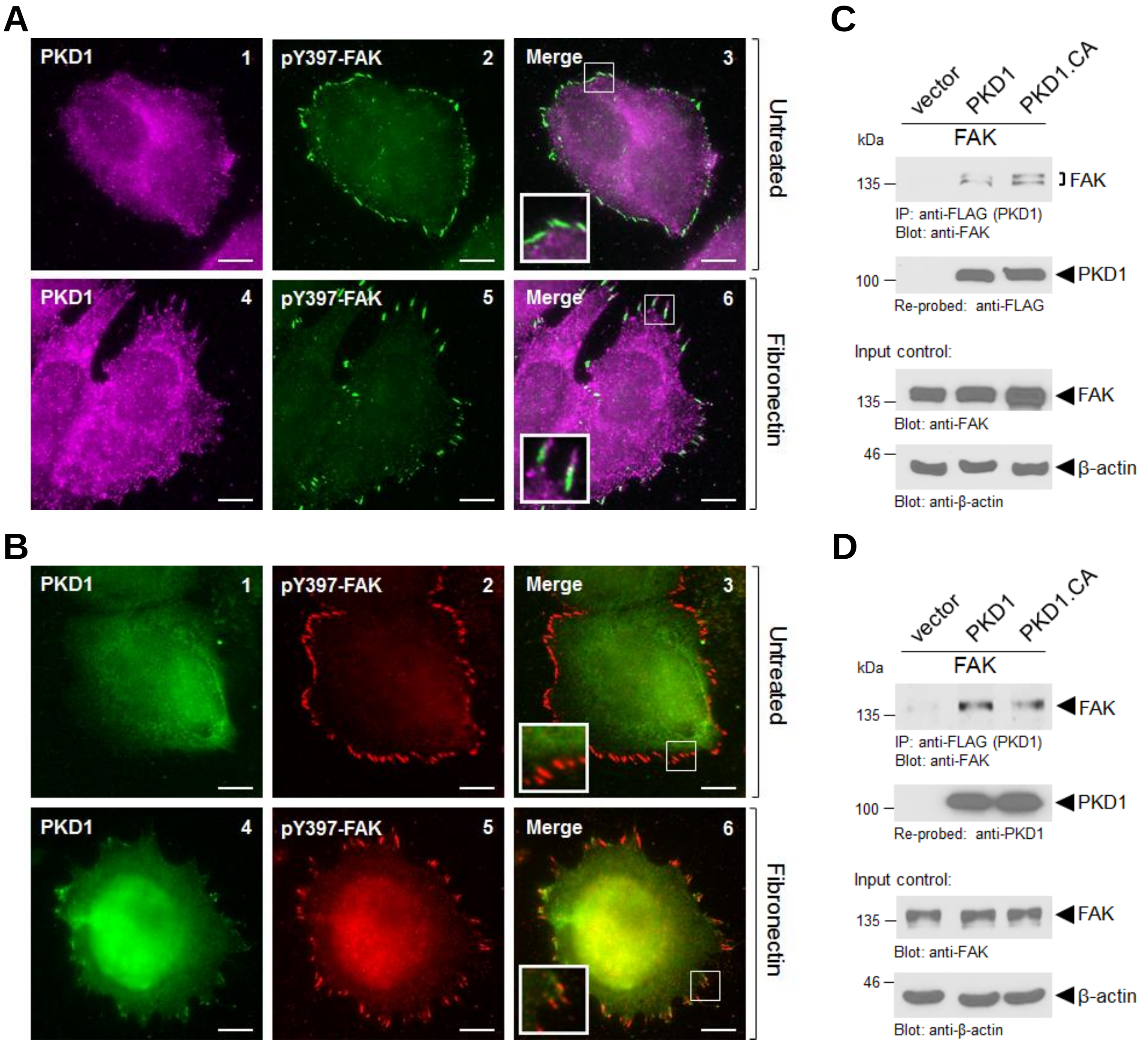

Supplemental Figure S1, relates to Figure 1: **Cellular localization of endogenous PKD1 and pY397-FAK, and co-immunoprecipitation of ectopically expressed PKD1 and FAK. A, B:** MCF-7 cells (0.10 x 10^6 cells /well, 24-well plate) were seeded on fibronectin-coated (2 µg/ml) glass coverslips and serum-starved for 16 hrs. Following serum-starvation, cells were stimulated with 10 µg/ml fibronectin or left untreated. After 30 minutes, cells were fixed and the localization of endogenous PKD1(Everest antibody in **A**; Abnova antibody in **B**) and pY397-FAK was determined by immunofluorescence analysis. Scale bars indicate 10 µm. Insets are 2.4-fold enhanced. **C, D:** Hek293T cells (0.5 x 10^6 cells/well, 6 well plate) in **C**, or NIH-3T3 cells (2 x 10^6 cells/10 cm dish) in **D,** were transfected with mCherry-FAK and vector control, FLAG-tagged wildtype PKD1 (PKD1) or FLAG-tagged constitutively-active PKD1 (PKD1.CA) as indicated. PKD1 was immunoprecipitated (anti-FLAG) and immunoprecipitates were analyzed for co-immunoprecipitated FAK. Samples were re-probed for overexpressed PKD1. Control Western blots of lysates were probed for FAK and β-actin expression.

Supplemental Figure S2, relates to Figure 2

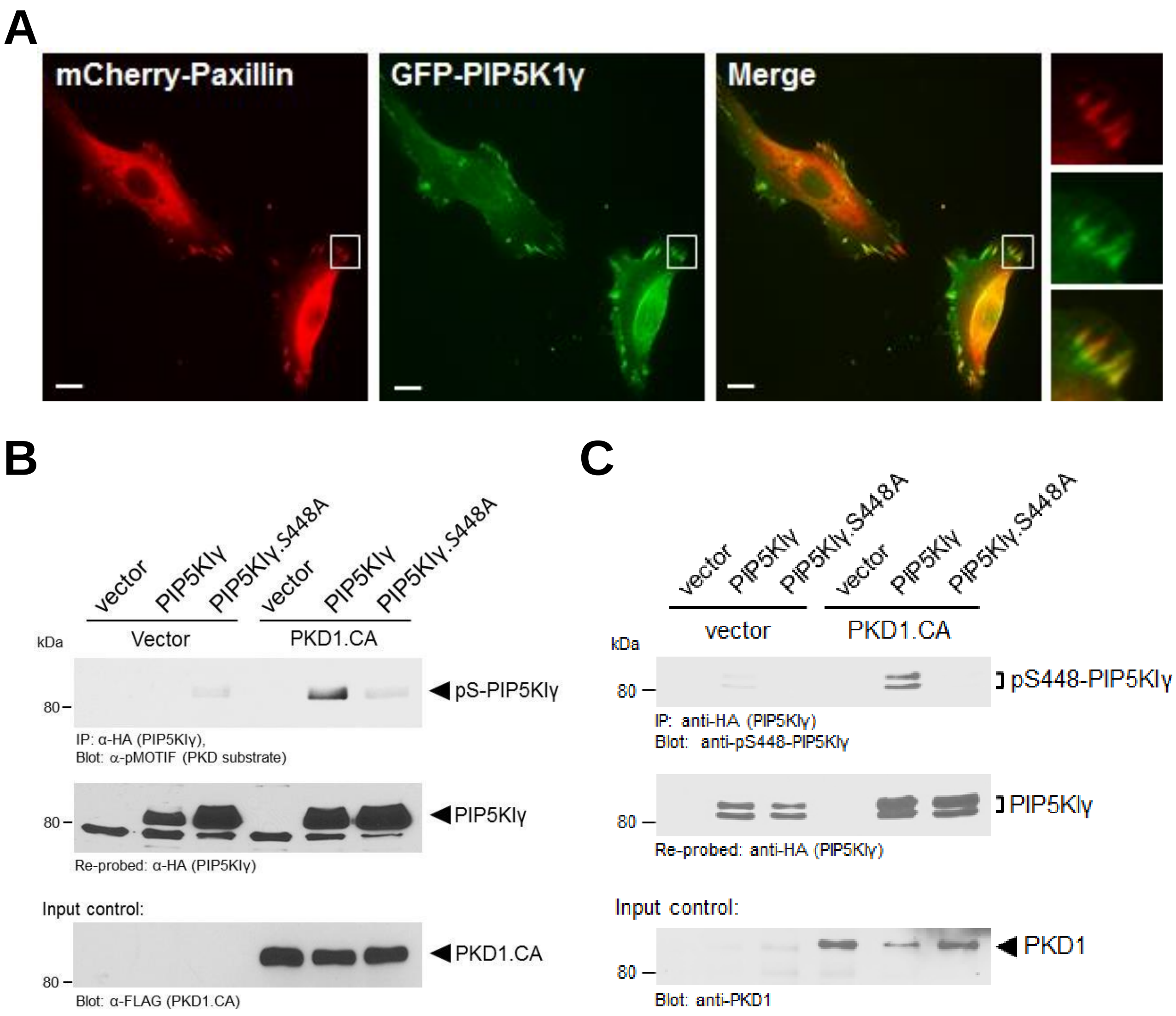

Supplemental Figure S2, relates to Figure 2: **PIP5KIγ localizes to focal adhesions and is phosphorylated by PKD1 at S448. A:** NIH-3T3 cells were co-transfected with mCherry-Paxillin and GFP-PIP5KIγ. Scale bars indicate 10 µm. The inset in is 6.5-fold enhanced on the right. **B:** HeLa cells (0.65 x 10^6 cells/6 cm dish) were co-transfected with vector and FLAG-tagged constitutively-active PKD1 (PKD1.CA) and vector, HA-tagged PIP5KIγ or PIP5KIγ.S448A as indicated. PIP5KIγ was immunoprecipitated (anti-HA) and assessed for PKD1-mediated phosphorylation using the pMOTIF antibody which recognizes phosphorylated PKD substrates. Immunoprecipitates were re-probed with anti-HA for equal loading of PIP5KIγ and PIP5KIγ.S448A. Lysates were evaluated for PKD1.CA expression (anti-FLAG). **C:** HeLa cells (0.25 x 10^6 cells/well, 6 well plate) were co-transfected with vector control or FLAG-tagged constitutively-active PKD1 (PKD1.CA) and vector, HA-tagged PIP5KIγ or PIP5KIγ.S448A, as indicated. PIP5KIγ was immunoprecipitated (anti-HA) and assessed for PKD1-mediated phosphorylation using a pS448-PIP5KIγ antibody. Immunoprecipitates were re-probed with anti-HA for equal loading of PIP5KIγ and PIP5KIγ.S448A. The lysates were evaluated for PKD1 expression (anti-PKD1).

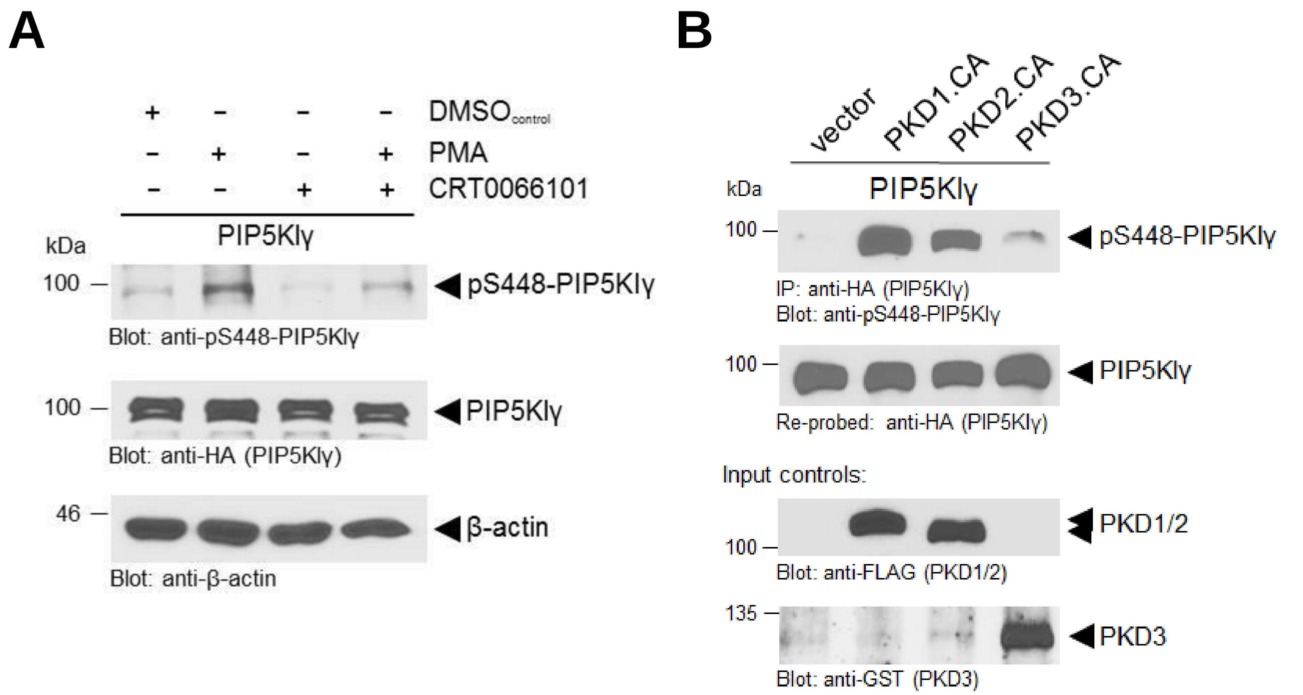

Supplemental Figure S3, relates to Figure 2: **PIP5KIγ is mainly phosphorylated by PKD1**. **A:** Hek293T cells (0.6 x 10^6 cells/well, 6 well plate) were transfected with HA-tagged PIP5KIγ, and stimulated with PMA (500 nM, 15 min), CRT0066101 (2.5 µM, 60 min) or DMSO control as indicated. Cell lysates were probed for PIP5KIγ phosphorylation (anti-pS448-PIP5KIγ) and re-probed for total PIP5KIγ (anti-HA). Control blots were probed for β-actin. **B:** Hek293T cells (0.5 x 10^6 cells/well, 6 well plate) were co-transfected with HA-tagged PIP5KIγ along with vector control, FLAG-tagged constitutively-active PKD1 (PKD1.CA), FLAG-tagged constitutively-active PKD2 (PKD1.CA), or GST-tagged constitutively-active PKD3 (PKD3.CA). An anti-HA antibody was used to immunoprecipitate PIP5KIγ from cells. Immunoprecipitates were subjected to SDS PAGE, and assessed using a pS448-PIP5KIγ antibody. Samples were counterstained for total PIP5KIγ using an anti-HA antibody. Control blots were performed for PKD1/2 (anti-FLAG), PKD3 (anti-GST) and β-actin expression.

Supplemental Figure S4, relates to Figure 3

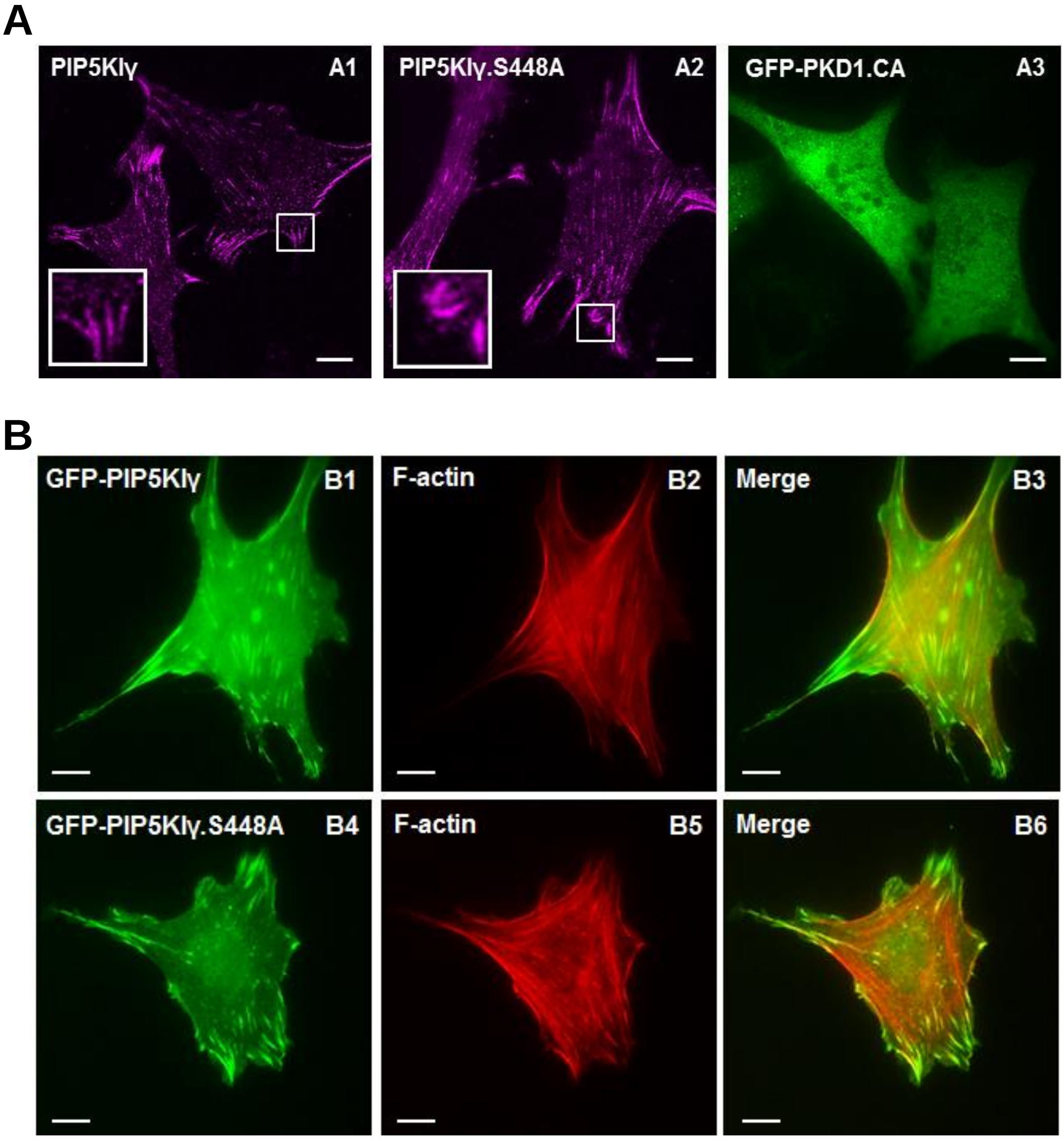

Supplemental Figure S4, relates to Figure 3: **Cellular localization of PIP5KIγ and PIP5KIγ.S448A. A:** Controls for Figure 3A. NIH-3T3 cells expressing HA-tagged PIP5KIγ or PIP5KIγ.S448A or GFP-tagged active PKD1 (GFP-PKD1.CA) were seeded (0.05 x 10^6 cells/well, 24 well plate) on fibronectin-coated (2 µg/ml) glass coverslips. Following fixation, immunofluorescence analysis was used to determine the localization of PIP5KIγ and GFP-PKD1. Scale bars indicate 10 µm. **B:** NIH-3T3 cells co-expressing GFP-PIP5KIγ or GFP-PIP5KIγ.S448A and RFP-LifeACT (to label F-actin structures) were seeded on fibronectin-coated (2 µg/ml) glass coverslips (0.05 x 10^6 cells/well, 24 well plate). Following fixation, cells were analyzed by immunofluorescence. Scale bars indicate 10 µm.

Supplemental Figure S5, relates to Figure 3

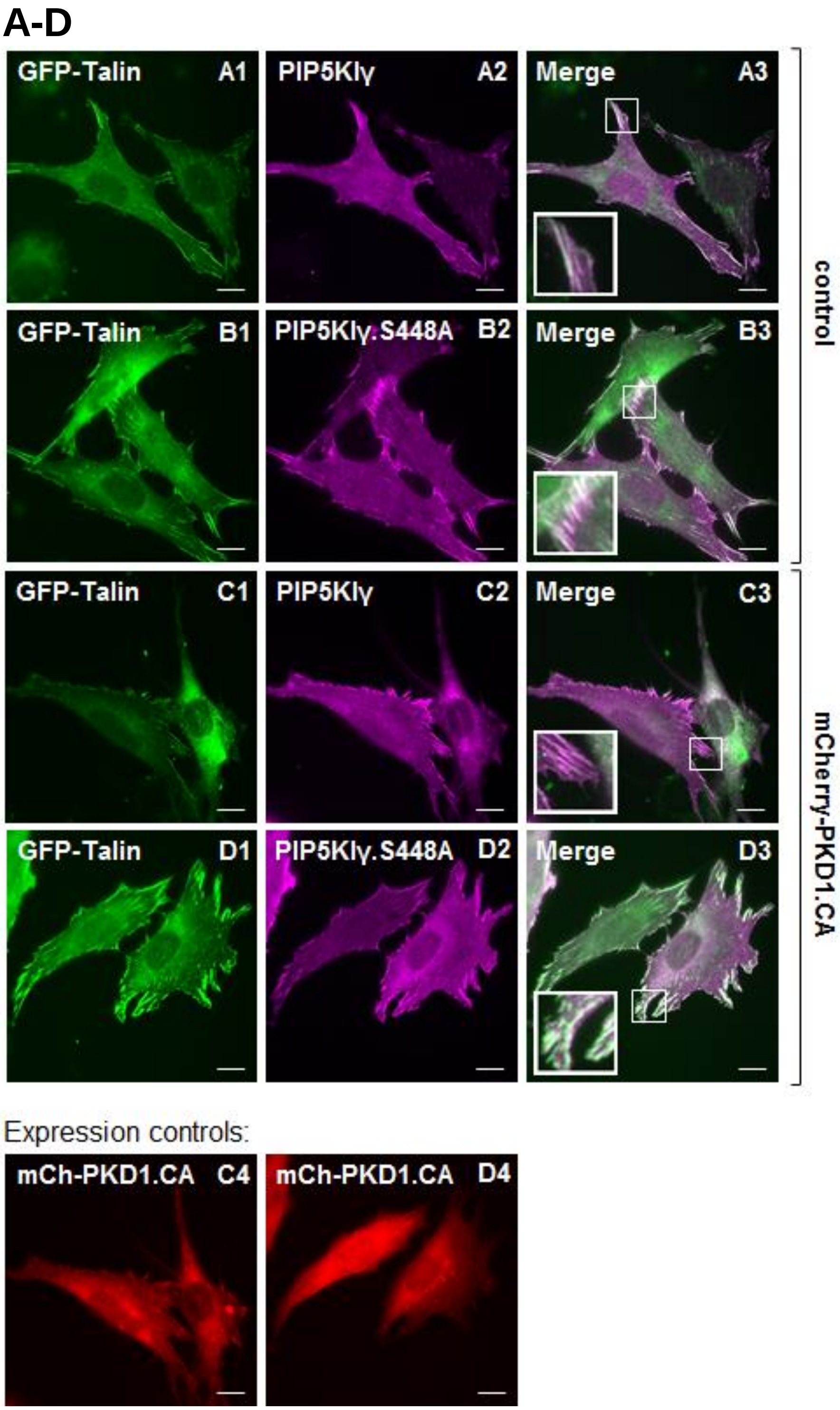

Supplemental Figure S5, relates to Figure 3: **S448 phosphorylation inhibits interaction of PIP5K1γ and Talin. A-D:** NIH-3T3 cells expressing mCherry-PKD1.CA, GFP-Talin with HA-tagged PIP5KIγ or PIP5KIγ.S448A were seeded on fibronectin-coated (2 µg/ml) glass coverslips. Localization of Talin and PIP5KIγ was determined by immunofluorescence analysis. Scale bars indicate 10 µm. Insets are 2.6-fold enhanced.

Supplemental Figure S6, relates to Figure 4

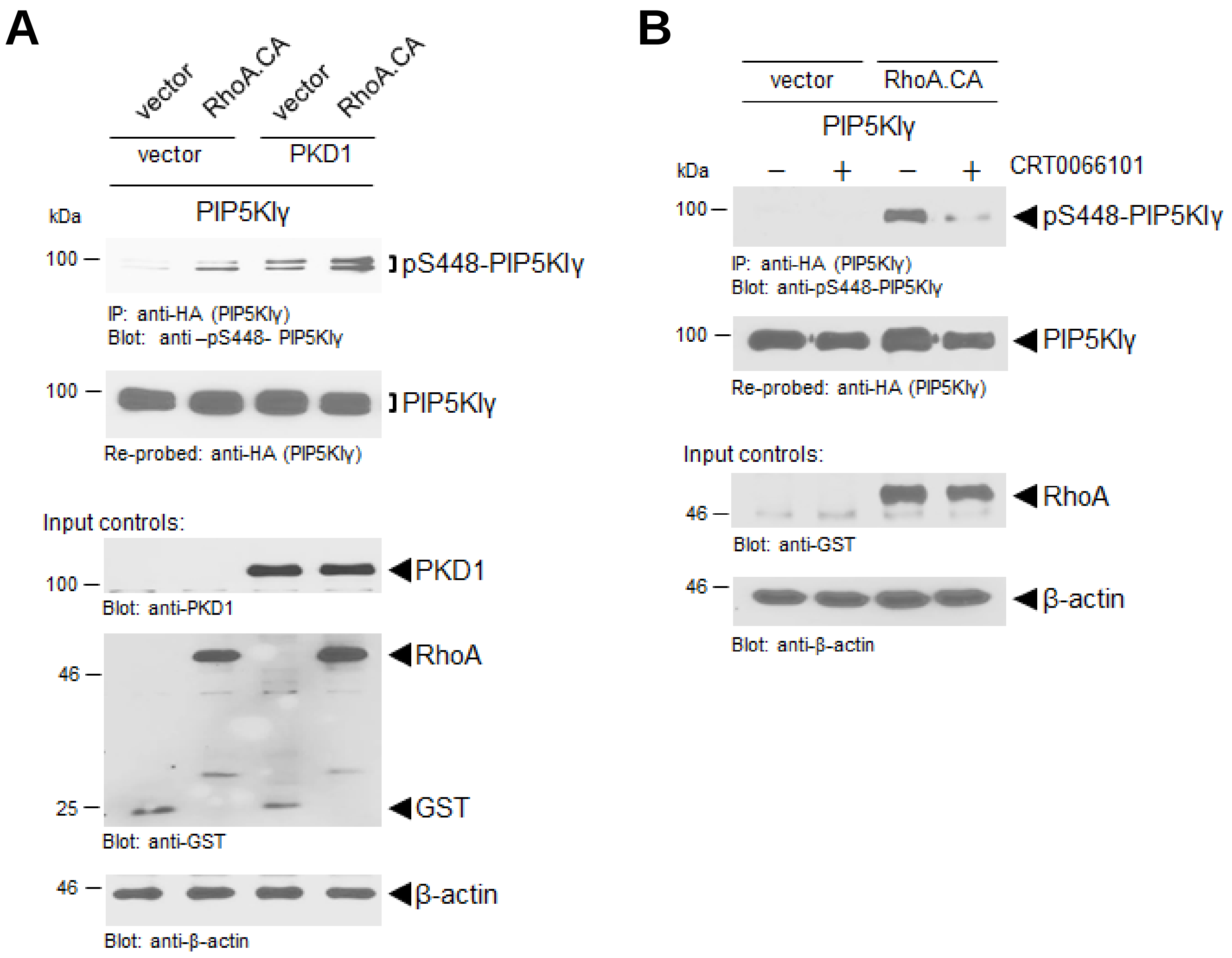

Supplemental Figure S6, relates to Figure 4: **PKD1-mediated phosphorylation of S448 occurs in response to Fibronectin-RhoA signaling. A:** HeLa cells (0.25 x 10^6 cells/well, 6 well plate) were co-transfected with HA-tagged PIP5KIγ, vector or FLAG-tagged PKD1 and vector or GST-tagged active RhoA (RhoA.CA), as indicated. PIP5KIγ was immunoprecipitated (anti-HA) and analyzed using pS448-PIP5KIγ antibody. Samples were re-probed with anti-HA to determine PIP5KIγ loading. Control blots were probed for PKD1, RhoA.CA (anti-GST) and β-actin expression. **B:** HeLa cells (0.25 x 10^6 cells/well, 6 well plate) were co-transfected with HA-tagged PIP5KIγ and vector control or GST-tagged active RhoA (RhoA.CA). Cells were stimulated with CRT0066101 (500 nM, 16 hours) or left untreated as indicated. PIP5KIγ was immunoprecipitated (anti-HA) and analyzed using the pS448- PIP5KIγ antibody. Immunoprecipitates were re-probed for total PIP5KIγ (anti-HA). Control blots were probed for RhoA.CA (anti-GST) and β-actin expression.

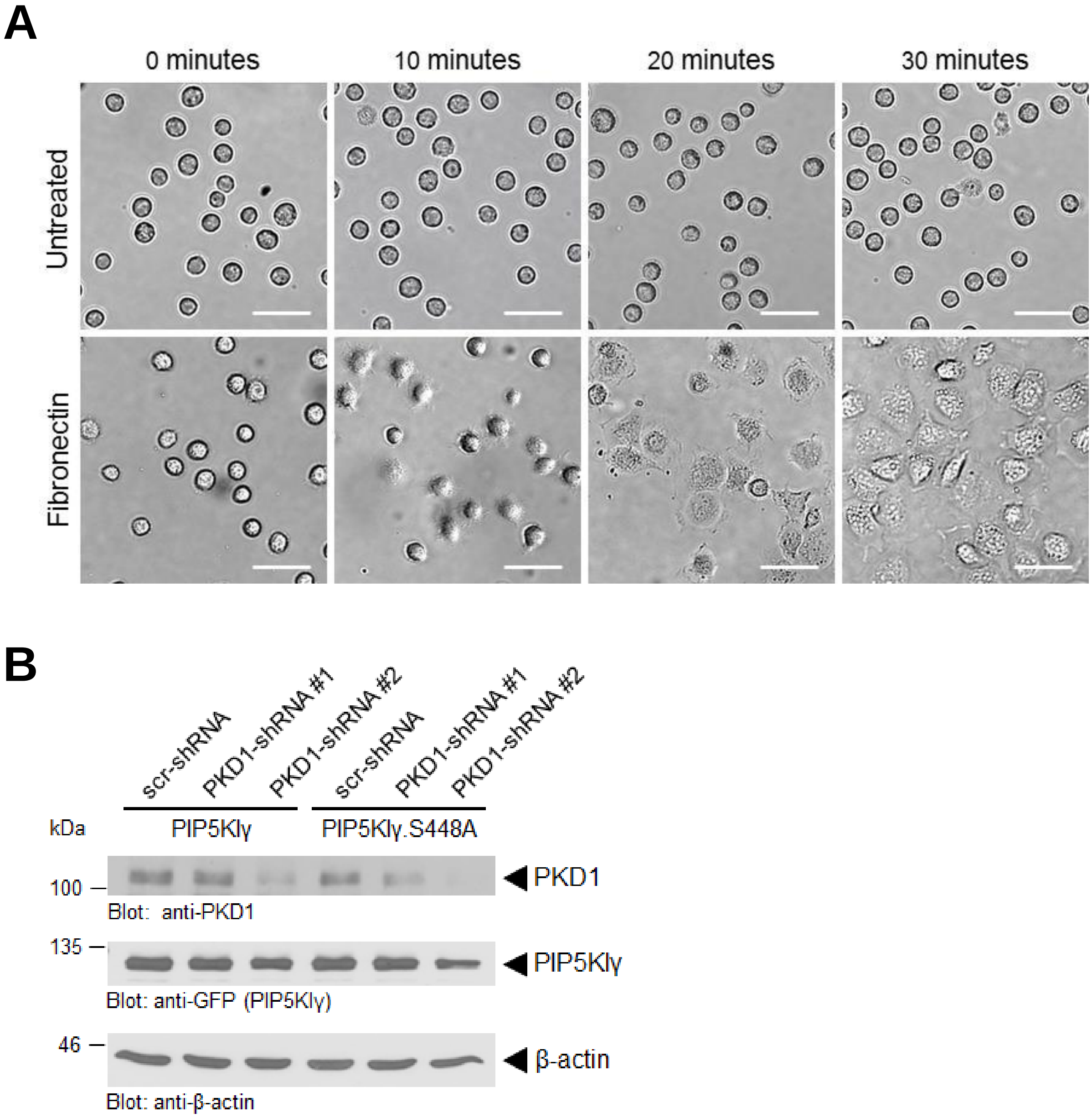

Supplemental Figure S7, relates to Figure 5: **Attachment of NIH-3T3 cells to fibronectin. A:** NIH-3T3 cells (0.05x 10^6 cells/well, 24 well plate) were plated on non-coated or fibronectin-coated (10 µg/ml) glass coverslips. Bright field images were acquired at the times indicated to determine the number of cells attached. The bar indicates 50 µm. **B:** Control blots for Figures 5C and 5D. Cell lysates were evaluated by Western blotting for the expression of PIP5KIγ (anti-GFP), PKD1 and β-actin.
